# An observational study of patient versus parental perceptions of health-related quality of life in children and adolescents with a chronic pain condition: who should the clinician believe?

**DOI:** 10.1186/1477-7525-10-85

**Published:** 2012-07-23

**Authors:** Thomas R Vetter, Cynthia L Bridgewater, Gerald McGwin

**Affiliations:** 1 Department of Anesthesiology, University of Alabama School of Medicine, 619 19th Street South, JT-862, Birmingham, AL, 35249-6810, USA; 2 Department of Nursing, Children’s Hospital of Alabama, 1600 7th Avenue South, Birmingham, AL, 35233, USA; 3 Department of Epidemiology, School of Public Health, University of Alabama at Birmingham, 1922 7th Ave. South, Suite 120, Birmingham, AL, 35294-0022, USA

**Keywords:** Health-related quality of life, Chronic pain, Pediatric, Children, Adolescents, Proxy-report, Child-parent agreement

## Abstract

**Background:**

Previous pediatric studies have observed a cross-informant variance in patient self-reported health-related quality of life (HRQoL) versus parent proxy-reported HRQoL. This study assessed in older children and adolescents with a variety of chronic pain conditions: 1) the consistency and agreement between pediatric patients’ self-report and their parents’ proxy-report of their child’s HRQoL; 2) whether this patient-parent agreement is dependent on additional demographic and clinical factors; and 3) the relationship between pediatric patient HRQoL and parental reported HRQoL.

**Methods:**

The 99 enrolled patients (mean age 13.2 years, 71% female, 81% Caucasian) and an accompanying parent completed the PedsQL^TM^ 4.0 and 36-Item Short-Form Health Survey Version 2 (SF-36v2) at the time of their initial appointment in a pediatric chronic pain medicine clinic. Patients’ and parents’ total, physical, and psychosocial HRQoL scores were analyzed via an intra-class correlation coefficient, Spearman’s correlation coefficient, Wilcoxon signed rank test, and Bland-Altman plot. A multivariable linear regression model was used to evaluate the association between clinical and demographic variables and the difference in patient and proxy scores for the Total Scale Score on the PedsQL™.

**Results:**

With the exception of the psychosocial health domain, there were no statistically significant differences between pediatric patients’ self-report and their parents’ proxy-report of their child’s HRQoL. However, clinically significant patient-parent variation in pediatric HRQoL was observed. Differences in patient-parent proxy PedsQL™ Total Scale Score Scores were not significantly associated with patient age, gender, race, intensity and duration of patient’s pain, household income, parental marital status, and the parent’s own HRQoL on the SF-36v2. No significant relationship existed among patients’ self-reported HRQoL (PedsQL™), parental proxy-reports of the child’s HRQoL, and parents’ own self-reported HRQoL on the SF-36v2.

**Conclusions:**

We observed clinically significant variation between pediatric chronic pain patients’ self-reports and their parents’ proxy-reports of their child’s HRQoL. While whenever possible the pediatric chronic pain patient’s own perspective should be directly solicited, equal attention and merit should be given to the parent’s proxy-report of HRQoL. To do otherwise will obviate the opportunity to use any discordance as the basis for a therapeutic discussion about the contributing dynamic with in parent-child dyad.

## Background

Health-related quality of life (HRQoL) is an essential element in the assessment of chronic disease. It has been advocated that the measurement of HRQoL should be routine in pediatric outcomes research and clinical practice [1-5]. Specifically, the Pediatric Initiative on Methods, Measurement, and Pain Assessment in Clinical Trials (PedIMMPACT) [3] and the Patient-Reported Outcome Measurement Information System (PROMIS) have advocated the measurement of children's self-reported health, illness, well-being, and quality of life, including with a chronic pain conditions [2,6,7].

For older children and adolescents, self-reports of such health information are reportedly of primary interest and meaning [3]. Nevertheless, seeking pediatric care is at least equally driven by the parent’s perspective of the child’s illness, and clinical decision-making is compelled to consider the parental proxy-report of pediatric HRQoL [8-12]. However, previous pediatric studies have observed an imperfect concordance or a cross-informant variance in patient self-reported HRQoL versus parent proxy-reported HRQoL [8,13,14]. This discordance has been observed not only in healthy subjects [15,16] and in a community adolescent sample [17], but also in patients with a psychiatric disorder [18], migraine headache [19,20], inflammatory bowel disease [21], functional abdominal pain [22], juvenile arthritis [11], cancer [23,24], and sickle cell disease [25]. However, to date, no study has examined the relationship between patient self-reported and parent proxy-reported HRQoL in individuals referred to pediatric chronic pain medicine program. Furthermore, the factors that are associated with this patient-parent discordance have not been identified, and no recommendations have been made as to how to clinically reconcile such patient-parent discordance.

This study was thus undertaken to assess in older children and adolescents with a variety of chronic pain conditions, who were referred to such a subspecialty clinic: 1) the consistency and agreement between pediatric patients’ self-report and their parents’ proxy-report of their child’s HRQoL; 2) the degree to which this patient-parent agreement is dependent on additional demographic and clinical factors; and 3) the relationships among pediatric patients’ self-reported HRQoL, parents’ proxy-reports of their child’s HRQoL, and parents’ self-reports of their own respective HRQoL. Recommendations are made as to how to reconcile a differing perspective between the child and parent about a pediatric chronic pain patient’s HRQoL.

## Methods

### Setting and participants

Study participation was offered to 145 eligible patients, ranging between 8 years and 17 years of age, who were initially evaluated in an outpatient pediatric chronic pain medicine clinic, located at a free-standing children’s hospital, between May 2009 and December 2010. All patients were directly referred to this ambulatory clinic by their primary care physician or another subspecialist physician, with an existing chronic pain diagnosis.

Because of the low prevalence in the study population of persons of Hispanic or Latino origin (3.2%) and of a language other than English (e.g., Spanish) being spoken at home (3.2%) [26], patients in whose nuclear families English was not the primary or native language were excluded from this study. Patients suffering from severe cognitive dysfunction (i.e., mental retardation) and thus unable to complete the patient questionnaires were also excluded.

This study was approved by the Institutional Review Board of the University of Alabama at Birmingham and conducted with internationally recognized ethical standards. Of the 145 eligible patients, 99 (68%) agreed to participate and provided written parental consent and written patient assent. No data were collected on the reasons for study non-participation. However, the relatively low participation rate appeared due to frequently expressed parental time constraints and the already extensive evaluation performed on all new clinic patients. If both parents were available, one volunteered to serve as the sole study participant.

The majority of the 99 enrolled study participants were female (71%), early adolescents (mean age of 13.2years, SD of 2.4). Eighty-one percent were Caucasian and 17% African-American. The enrolled one Native American, one Asian-American, and one Hispanic patient/parent dyad were excluded from the regression analyses that included patient race. The composition of the 46 non-participants was 64% female and 79% Caucasian. Thus Caucasian males and their parents slightly less often agreed to participate.

The enrolled patients presented with a variety of primary chronic pain conditions: headache (21%); cervical, thoracic, lumbar, and/or sacral spine pain (19%); abdominal pain (18%); extremity or large joint pain (18%); fibromyalgia or a myofascial pain syndrome (15%); or peripheral neuropathic pain (including complex regional pain syndrome, CRPS, Types I and II) (8%). All study patients had experienced their presenting pain condition for more than one month (median duration of 15 months, interquartile range of 7 months-36 months).

### Study design

Enrolled patients and their parents completed the study questionnaires and health surveys at the time of their initial appointment in the pediatric chronic pain medicine clinic, but prior to being evaluated and treated by a pain medicine physician or any other clinic health care providers. The patient and the parent were consistently instructed by the study coordinator (C.L.B.) on how to complete the various measurement instruments and to complete them independently, so as to minimize any respondent cross-contamination. Patients and their parents were provided ample time and privacy to complete the study forms.

### Study measures

#### Patient health-related quality of life: Pediatric Quality of Life Inventory (PedsQL™) 4.0 Generic Core Scales

A recent evidence-based review of the various assessment tools for pediatric health-related quality of life and functional impairment examined 16 measures, including generic health-related scales, disease-specific quality of life scales, and functional impairment ratings [4]. Based upon published psychometric properties (three types of reliability and two types of validity), this workgroup classified 12 of the 16 measures, including the Pediatric Quality of Life Inventory (PedsQL™), as “well-established.”

The PedsQL™ (Mapi Research Institute, Lyon, France) is a valid and reliable, yet low respondent burden instrument that assesses patients’ and parents’ perceptions of generic health-related quality of life [27,28]. There are 23-items (each with a 0 = “Never” to 4 = “Almost Always” Likert scale) on PedsQL™ 4.0 Generic Core Scales. The PedsQL™ 4.0 Generic Core Scales generate a composite 0 (lowest health-related quality of life) to 100 (highest health-related quality of life) Total Scale Score [29]. A 0 to 100 subscale score can also be generated for each of the four domains (Physical Functioning, Emotional Functioning, Social Functioning, and School Functioning) on the PedsQL™ Generic Core Scales. The Physical Functioning domain subscale score is conventionally reported as the PedsQL™ Physical Health Summary Score. The PedsQL™ Psychosocial Health Summary Score equals the sum of the items divided by the number of items answered on the Emotional, Social, and School Functioning subscales [10]. There are developmentally appropriate, self-report versions of the PedsQL™ for the 8-12 and 13-18 year olds [30]. The parent proxy-version of the PedsQL™ has demonstrated adequate feasibility, reliability, and validity in parents recruited from general pediatric clinics, subspecialty clinics, and hospitals in which their children were being seen for well-child checks, mild acute illness, or chronic illness care [9]. The PedsQL™ was the most widely applied pediatric health-related quality of life measurement instrument in the MEDLINE database from 1966 to 2006 [31]. The accepted minimal clinically important difference (MCID) is 4.5 points for the PedsQL™ [29,32,33], the value that was applied here.

#### Pain intensity: Pediatric Pain Questionnaire (PPQ)

The Pediatric Pain Questionnaire (PPQ) is a patient self-reported and age-specific (child: 8-12 years; and adolescent: 13-18 years) pain assessment instrument [34,35]. The PPQ assesses the intensity, location(s) and other, more subjective affective characteristics of a patient’s pain [36,37]. The PPQ includes a 100 millimeter horizontal line (a Visual Analogue Scale, VAS) that is without numbers but ranges from 0 (anchored, depending on the age of the patient, either by a smiling carton face and “no hurt at all” or by “no pain, not hurting, no discomfort”) to 100 (anchored, depending on the age of the patient, either by a sad cartoon face and “hurting a whole lot” or by “severe pain, hurting a whole lot, very uncomfortable”). The PPQ assesses pain intensity at the present time (“now”) and at its worst in the past week. The PPQ has been shown to be a reliable and valid tool for measuring pediatric chronic pain intensity [36]. The utility of the PPQ was confirmed in a study of children and adolescents with chronic musculoskeletal pain associated with rheumatologic disease [38] and with sickle cell disease [39]. In this study we used the PPQ pain intensity score on presentation to the clinic. No other sensory or affective elements of the PPQ were utilized for this study.

#### Parent health-related quality of life: 36-item Short-Form Health Survey (SF-36)

In an effort to standardize clinical outcomes measurement, the adult Initiative on Methods, Measurement, and Pain Assessment in Clinical Trials (IMMPACT) identified six core outcome domains to be considered when designing chronic pain treatment efficacy and effectiveness trials [40]. The adult IMMPACT has recommended that the SF-36 be incorporated “as a generic measure of physical functioning because of the large amount of data available to permit comparisons among different disorders and treatments” [41]. The SF-36 was applied in a preliminary study of child self-reported quality of life versus parent self-reported quality of life in a group of healthy young children [15].

The 36-Item Short-Form Health Survey Version 2 (SF-36v2, QualityMetric Inc., Lincoln, RI) [42-44] was used to assess parents' own well-being. Currently the most widely used generic HRQoL measure in the world, the SF-36v2 has well-established validity and reliability [45,46]. The SF-36v2 includes eight subscales for physical functioning, physical role limitation, social role limitation, social functioning, mental health, energy/vitality, pain, and general health perception. Physical Health and Mental Health dimension scores, and a total score (equal to the mathematical average of its eight subscale scores) can be generated for the SF-36.

### Statistical analyses

Agreement between the patient self-reported and parent proxy-reported Total Scale Score and the Physical Health and Psychosocial Heath Summary Scores on the PedsQL™ was assessed using intra-class correlation coefficients (ICC) [47-49]. Pearson (parametric) and Spearman (non-parametric) correlation coefficients are interclass correlations in that they assess the correlation between two *different* variables (e.g., pain intensity and HRQoL). The ICC, as its name suggests, is an intra-class correlation and as such measures how members of the same group, or class, correlate with each other (e.g., paired parent-child ratings of a child’s HRQoL) [28]. As the group differences were not normally distributed, the PedsQL™ scores were analyzed with a Wilcoxon signed rank test. These sub-analyses were conducted for the total included and completed sample (N = 97) and separately for two patient age groups (8-12 years, N = 37 and 13-17 years, N = 60).

The frequency distribution of agreement was calculated between the patient self-reported and parent proxy-reported PedsQL™ Total Scale Scores, Physical Health Summary Scores and Psychosocial Heath Summary Scores, using the accepted minimal clinically important difference (MCID) of 4.5 points for the PedsQL™ as the cut-point. To evaluate any systematic tendency in the total study sample for parent proxy-reports to overestimate or underestimate health status compared to patient self-reports [24,48,50], Bland-Altman plots [51,52] were also generated for the PedsQL™ Total Scale Scores, Physical Health Summary Scores and Psychosocial Heath Summary Scores [14,53,54].

A multivariable linear regression model was used to evaluate within the entire study sample the association between patient age (years), patient gender (female or male), patient race (African-American or Caucasian), duration of patient’s pain (months), pain intensity (PPQ VAS), household income (<$25,000, $25,001-$50,000, $50,001-$75,000, $75,001-$100,000, or > $100,000), parental marital status (single, married, or divorced), and parental total SF-36 score *versus* the difference in the patient self-reported and parent-proxy scores for the Total Scale Score on the PedsQL™. A stepwise regression method, with entry criterion of p ≤ 0.05 and removal criterion of p ≥ 0.10, was used. Similar multivariable linear regression modeling has been applied to analzye the difference in parent-child dyad reports of HRQoL with cerebral palsy, epilepsy, and juvenile idiopathic arthritis [54-56]. However, as a sensitivity analysis, a multivariable logistic regression model was also performed to assess the association of the same eight independent variables with the binary presence of an absolute difference of ≥ 4.5 (the MCID) in the PedsQL™ Total Scale Score.

In addition to age, gender, and race, we chose the duration and intensity of the patient’s chronic pain as two common elements of chronic pain clinical variability. The parental total SF-36 score was chosen as a proxy measure of parental enmeshment, assuming a greater likelihood of parental excessive involvement or engrossment with greater concomitant parental dysfunction [57]. Household income and parental marital status were chosen based upon the Family Stress Model [58]. The Family Stress Model has been endorsed by American Academy of Pediatrics (AAP) and implicitly guided the AAP in generating its “Family Pediatrics: Report of the Task Force on the Family” [59]. The Family Stress Model is predicated upon the intrinsic interrelationship between socioeconomic status, family processes, and human development. The model acknowledges the strong correlation between lower socioeconomic status (with its attendant economic hardships) and disparities in the cognitive, social, emotional, and physical health and well-being of children, adolescents, and young adults [60].

Based upon previous published recommendations [8,13,15], we had planned *a priori* to include the category of the parental study participant/respondent (mother versus father) as a variable in our regression model. However, only 6 (6.1%) of the presently enrolled parental study participants and survey respondents were the patient’s father, a number too small to allow for a valid assessment of the effect of the parental respondent in patient-parent HRQoL agreement.

The relationship between patient self-reported HRQoL (PedsQL™ Total Scale Score and the Physical Health and Psychosocial Heath Summary Scores) and parental self-reported HRQoL (SF-36 Total and Physical Health and Mental Health dimension scores) was assessed using Spearman’s correlation coefficients, for the total included and completed sample (N = 96) and separately for the two patient age groups (8-12 years, N = 37 and 13-17 years, N = 59).

The relationship between parent proxy-reported HRQoL (PedsQL™ Total Scale Score and the Physical Health and Psychosocial Heath Summary Scores) and parental self-reported HRQoL (SF-36 Total and Physical Health and Mental Health dimension scores) was also assessed using Spearman’s correlation coefficients, for the total included and completed sample (N = 98) and separately for the two patient age groups (8-12 years, N = 37 and 13-17 years, N = 61).

For the multiple correlations and comparisons between the patient self-reported and parent proxy-reported HRQoL scores, a more conservative p-value of < 0.01 was considered significant, so as to reduce the risk of a Type I error (α = 0.01). Applying this more conservative alpha may have reduced the statistical power of the related test statistic and increased the risk of a Type II error. All statistical analyses were performed using IBM® SPSS® (Version 19.0). Bland-Altman plots were generated using MedCalc® (Version 11.6.1.0).

## Results

### Range of measurements

Table 1 presents the mean and standard deviation values for the patient self-reported and parent proxy-reported PedsQL™ scores. The patient self-reported and parent proxy-reported HRQoL scores were generally not normally distributed and thus were subjected to non-parametric statistics. The mean patient self-reported and parent proxy-reported Total Scale Score and the Physical Health and Psychosocial Heath Summary Scores on the PedsQL™ were also generally low.

**Table 1:** **Patient self-reported and parent proxy-reported health-related quality of life scores on the PedsQL**^**TM**^

**Scale**	**Total sample (N=97)**	**8-12 year olds (N=37)**	**13-18 year olds (N=60)**	
Total Score				
Patient	Mean (SD)	54.4 (20.2)	58.3 (20.4)	51.8 (19.9)
	Median (IQR)	54.3 (36.9-69.0)	58.7 (45.6-74.2)	53.3 (35.0-64.1)
Parent	Mean (SD)	50.1 (18.6)	53.8 (19.9)	48.2 (17.5)
	Median (IQR)	46.0 (34.8-63.2)	51.0 (41.0-74.0)	44.0 (33.5-60.0)
		p=0.083	p=0.17	p=0.33
Physical Health Summary Score				
Patient	Mean (SD)	44.4 (24.4)	47.3 (25.0)	42.6 (24.1)
Median (IQR)	43.7 (27.1-62.5)	43.7 (34.4-64.0)	40.5 (25.3-60.2)	
Parent	Mean (SD)	44.5 (23.2)	49.0 (23.0)	41.7 (23.2)
Median (IQR)	42.2 (27.2-63.2)	46.8 (31.2-66.2)	37.5 (21.9-62.5)	
		p=0.92	p=0.97	p=0.95
Psychosocial Health Summary Score				
Patient	Mean (SD)	57.7 (20.2)	60.7 (21.6)	55.8 (19.2)
Median (IQR)	58.3 (44.2-73.3)	61.7 (45.8-77.5)	57.5 (40.0-73.3)	
Parent	Mean (SD)	52.7 (17.9)	56.2 (20.0)	50.5 (16.3)
Median (IQR)	49.2 (40.0-63.3)	53.3 (42.5-70.0)	48.3 (40.0-59.6)	
		p=0.008	p=0.22	p=0.016

SD: standard deviation; IQR: interquartile range.

### Consistency and agreement between patient self-reported and parent proxy-reported HRQoL

#### Correlation consistency

Table 2 presents the intra-class correlation coefficients between the patient self-reported and the parent proxy-reported Total Scale Score and the Physical Health and Psychosocial Heath Summary Scores on the PedsQL™. The HRQoL score consistency between study patients and their parents was significant across the entire patient age range and within the two patient age strata (8-12 years, 13-17 years). However, none of the intra-class correlation coefficients were ≥ 0.70 and thus considered strong.

**Table 2:** **Correlations between patient self-reported and the parent proxy-reported HRQoL scores on the PedsQL**^**TM**^

**Scale**	**Intra-class correlation coefficients (95% CI)**		
	**Total sample (N=96)**	**8-12 year olds (N=37)**	**13-18 year olds (N=59)**
Total Score	0.61 (0.47, 0.72)	0.58 (0.32, 0.76)	0.62 (0.43, 0.75)
	p<0.001	p<0.001	p<0.001
Physical Health Summary Score	0.59 (0.44, 0.71)	0.46 (0.17, 0.68)	0.67 (0.50, 79)
	p<0.001	p=0.002	p<0.001
Psychosocial Health Summary Score	0.62 (0.48, 0.73)	0.63 (0.38, 0.79)	0.61 (0.42, 0.75)
	p<0.001	p<0.001	p<0.001

#### Agreement between HRQoL scores

Table 1 presents the median and interquartile range values for the patient self-reported and the parent proxy-reported PedsQL™ Total Scale Scores, PedsQL™ Physical Health Summary Scores, and PedsQL™ Psychosocial Health Summary Scores. With the exception of the Psychosocial Health Summary Score for the total study sample (p = 0.008), there was no statistically significant difference (p < 0.01) between the patient self-reported and the parent proxy-reported HRQoL scores. Despite this lack of significance, there was a pattern of parents reporting lower scores than patients.

**Figure 1:**
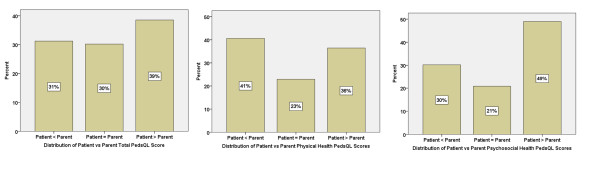
**Distribution of differences in patient self-reported versus parent proxy-reported PedsQL™ scores.** Agreement defined as an absolute PedsQL™ score difference of less than 4.5 (the minimal clinically important difference, MCID, for the PedsQL™). Patient<Parent and Patient>Parent defined as PedsQL™ score difference of greater than or equal to 4.5.

**Figure 2:**
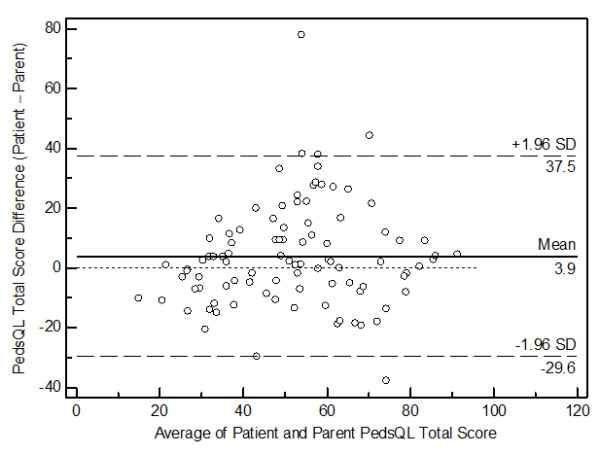
Bland-Altman plot for the PedsQL™ Total Scores.

**Figure 3:**
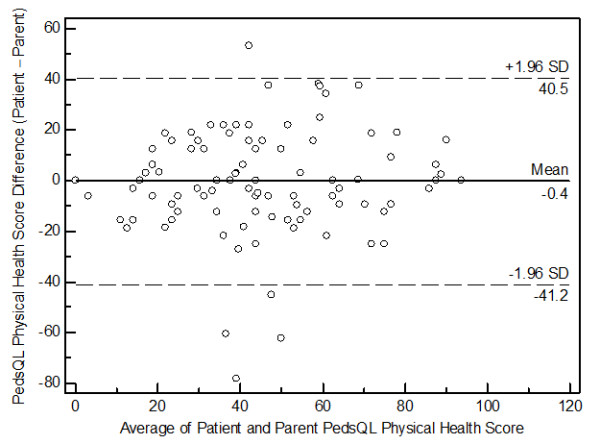
Bland-Altman plot for the PedsQL™ Physical Health Summary Scores.

**Figure 4:**
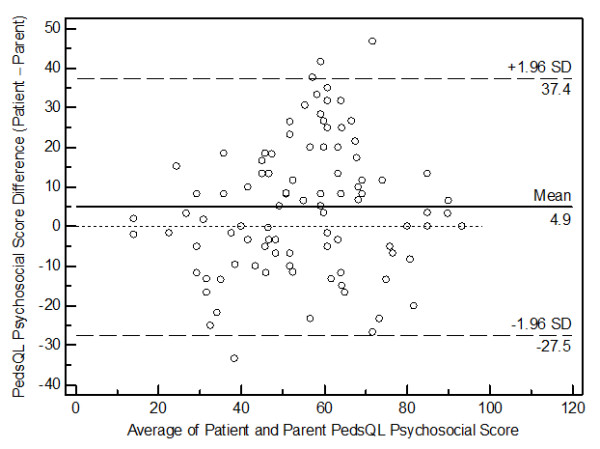
Bland-Altman plot for the PedsQL™ Psychosocial Health Summary Scores.

Agreement across the entire study sample between the patient self-reported versus the parent proxy-reported PedsQL™ scores (defined as an absolute score difference of less than 4.5, the MCID for the PedsQL™) was only 30% for the Total Score, 23% for the Physical Health Summary Score, and 21 % for the Psychosocial Heath Summary Scores (Figure 1). Furthermore, the Bland-Altman plots for the PedsQL™ Total Scale Scores (Figure 2), the PedsQL™ Physical Health Summary Scores (Figure 3), and the PedsQL™ Psychosocial Health Summary Scores (Figure 4) revealed a significant amount of variation (bias), with 95% limits of agreement ranging from −41.2 to 40.5, indicating relatively poor agreement between patient self-reported versus parent proxy-reported HRQoL. While the Physical Health Summary Scores demonstrated fairly uniform dispersion, the Total and Psychosocial Scores tended to indicate biased agreement at the extremes of the distribution.

### Predictors of patient-parent agreement about HRQoL

None of these eight independent variables (age, gender, race, duration of chronic pain duration, chronic pain intensity, household income, parental marital status, and parental total SF-36v2 score) was consistently significantly associated with the absolute difference between the patient self-reported and the parent proxy-reported Total Scale Score on the PedsQL™. A sensitivity analysis using a logistic regression model also revealed none of these eight independent variables to be significantly associated with the presence of an absolute difference ≥ 4.5 (the MCID) in the PedsQL™ Total Scale Score.

### Relationship between patient self-reported HRQoL and parent self-reported HRQoL

Table 3 presents the Spearman’s correlation coefficients between the enrolled patients’ self-reported HRQoL (PedsQL™ Total Scale Score and the Physical Health and Psychosocial Heath Summary Scores) and their parents’ own self-reported HRQoL (SF-36 Total and Physical Health and Mental Health dimension scores), for the total sample and separately for the two patient age groups (8-12 years and 13-17 years). No significant relationship (applying the *a priori* more conservative α of 0.01) was observed between these two health status measures.

**Table 3:** **Correlations between patients’ self-reported HRQoL scores on the PedsQL**^**TM**^**and parent’s self-reported HRQoL on SF-36v2**

**Scale**	**Spearman correlation coefficients (95% CI)**		
	**Total sample (N=97)**	**8-12 year olds (N=37)**	**13-18 year olds (N=60)**
PedsQL^TM^ Total Score	0.24 (0.04, 0.44)	0.27 (−0.04, 0.58)	0.23 (−0.03, 0.49)
*versus* SF-36 Total Score	p=0.16	p=0.11	p=0.078
PedsQL^TM^ Physical Health Summary Score	0.12 (−0.09, 0.34)	−0.12 (−0.46, 0.22)	0.30 (0.03, 0.56)
*versus* SF-36 Physical Health Score	p=0.23	p=0.49	p=0.21
PedsQL^TM^ Psychosocial Summary Score	0.20 (0.01, 0.4)	0.12 (−0.20, 0.43)	0.29 (0.04, 0.54)
*versus* SF-36 Mental Health Score	p=0.045	p=0.50	p=0.024

p < 0.01 considered significant due to *a priori* correction of alpha.

HRQoL: health-related quality of life.

PedsQL^TM^: Pediatric Quality of Life Inventory 4.0 Generic Core Scales.

SF-36v2: 36-Item Short-Form Health Survey Version 2.

### Relationship between parent proxy-reported HRQoL and parental self-reported HRQoL

Table 4 presents the Spearman’s correlation coefficients between the enrolled parent proxy-reported HRQoL (PedsQL™ Total Scale Score and the Physical Health and Psychosocial Heath Summary Scores) and these parents’ own self-reported HRQoL (SF-36 Total and Physical Health and Mental Health dimension scores), for the total sample and separately for the two patient age groups (8-12 years and 13-17 years). Applying an *a priori* more conservative α of 0.01, there was a significant relationship between the parent proxy-reported PedsQL™ Psychosocial Heath Summary Scores and the parents’ own self-reported SF-36 Mental Health dimension scores, for the total sample and separately for the older patient age groups (13-17 years).

**Table 4:** **Correlations between parents proxy-reported HRQoL scores on the PedsQL**^**TM**^**and parent’s self-reported HRQoL on SF-36v2**

**Scale**	**Spearman correlation coefficients (95% CI)**		
	**Total sample (N=98)**	**8-12 year olds (N=37)**	**13-18 year olds (N=61)**
PedsQL^TM^ Total Score	0.21 (0.02, 0.40)	0.11 (−0.22, 0.42)	0.26 (0.004, 0.48)
*versus* SF-36 Total Score	p=0.035	p=0.52	p=0.046
PedsQL^TM^ Physical Health Summary Score	0.15 (−0.05, 0.34)	0.03 (−0.30, 0.35)	0.24 (−0.02, 0.46)
*versus* SF-36 Physical Health Score	p=0.14	p=0.86	p=0.065
PedsQL^TM^ Psychosocial Summary Score	0.31 (0.12, 0.48)	0.28 (−0.05, 0.55)	0.36 (0.12, 0.56)
*versus* SF-36 Mental Health Score	p=0.002	p=0.09	p=0.004

p<0.01 considered significant due to *a priori* correction of alpha.

HRQoL: health-related quality of life.

PedsQL^TM^: Pediatric Quality of Life Inventory 4.0 Generic Core Scales.

SF-36v2: 36-Item Short-Form Health Survey Version 2.

## Discussion

Our findings indicate that in children and adolescents with a variety of chronic pain conditions referred to a subspecialty clinic: 1) there is clinically significant variation and some minimal clinically important differences between pediatric patients’ self-report and their parents’ proxy-report of their child’s HRQoL; 2) the patient-proxy differences (patient score minus proxy score) for the Total Scale Score on the PedsQL™ are not dependent on common demographic or clinical factors; and 3) while no significant relationship exists between patients’ self-reported HRQoL (PedsQL™ Total Scale Score and the Physical Health and Psychosocial Heath Summary Scores) and their parents’ own self-reported HRQoL (SF-36 Total and Physical Health and Mental Health dimension scores), there is a significant relationship between the parent proxy-reported PedsQL™ Psychosocial Heath Summary Scores and the parents’ own self-reported SF-36 Mental Health dimension scores. These collective findings are surprising and counter-intuitive—given Varni’s biobehavioral model of pediatric chronic pain, as well as Palermo and Chambers’ integrative model of pediatric chronic pain, both of which emphasize parental and environmental contributing factors, in addition to a strong interactive element in the parent-child dyad [61,62].

Previous studies of parent-child perceptions of HRQoL have applied various statistical methods, resulting in potentially artefactually conflicting results and conclusions [15]. Specifically, parent-child agreement should ideally be assessed at the level of the individual by intra-class correlation (rather than the more frequently applied Pearson’s or Spearman’s correlation) and the level of the group by comparison of means (or medians if non-parametric data) [8,63]. Additional insight into the relationship between self-reported and proxy-reported HRQoL can be provided by a Bland-Altman plot [51,52]. Recent studies of pediatric patients with serious physical trauma [53], with asthma [64], and after liver transplantation [14] also applied Bland-Altman plots to assess the agreement between parent and child reports of HRQoL. All three methods of assessing parent-child HRQoL agreement were applied here, to analyze data collected with PedsQL^TM^ 4.0 and SF-36v2 generic health status measures. It is perhaps important to note that no one of these methods is superior in all settings; rather each has unique properties that address specific aspects of agreement. Thus, future research should utilize an array of statistical tools in order to provide a comprehensive assessment of parent-child agreement.

Despite no consistent statistically significant difference in the median patient self-reported versus parent proxy-reported PedsQL™ Total Scale Scores, PedsQL™ Physical Health Summary Scores, and PedsQL™ Psychosocial Health Summary Scores (Table 1), we observed a pattern of parents reporting lower mean HRQoL scores than their children (greater than the MCID of 4.5) [29,32,33], especially for the Psychosocial Health Summary Scores. It would thus appear that parents overestimate the adverse effect of chronic pain in their children. A similar parental perception of lower PedsQL™ scores compared to their children (mean scores of 70 and 78, respectively) has been observed with functional abdominal pain [22]. This perception among parents of their child’s chronic pain condition having a greater adverse effect may be based upon externally visible and wearisome signs and symptoms of disease or sickness (e.g., frequent reports of pain, recurrent health care visits, greater school absenteeism, less peer and family interaction, reduced extracurricular participation—including physical activity), which can be perceived as less problematic or troublesome by the child (patient). This would be especially the case when a non-life threatening chronic pain condition affords the older child and adolescent the opportunity to avoid schoolwork, extracurricular events, and peer-pressure, but instead to take refuge in the home setting.

Furthermore, the Bland-Altman plots for the present PedsQL™ Total Scale Scores, the PedsQL™ Physical Health Summary Scores, and the PedsQL™ Psychosocial Health Summary Scores revealed a significant amount of variation (bias), indicating poor agreement between patient self-reported versus parent proxy-reported HRQoL. A similar wide variation in the 95% limits of agreement on Bland-Altman plots has been reported using the Child Health Questionnaire in adolescents and their mothers after pediatric liver transplant [14] and using the PedsQL^TM^ in children with cerebral palsy and their parents [54]. While this poor agreement may seem in conflict with the interpretations for the other statistical measures (e.g., ICCs indicating moderate agreement), as noted above, it is important to keep in mind that each of these measures addresses a specific, albeit related, aspect of agreement. Thus, consistency across measures is not a given, each measure provides unique insight and should be interpreted independently.

In our cohort of children and adolescents, with a wide variety of chronic pain conditions, there was a moderate patient-parent agreement (ICC ≈ 0.60) for overall health status and for the physical and psychosocial health domains on the PedsQL^TM^ across the entire age spectrum, as well as in the younger (8-12 year) and older (13-17 year) age groups. The present ICC values were greater than those observed for the PedsQL^TM^ in healthy younger children (0.02-0.23) [15], perhaps reflecting a greater level of parental interaction and communication in children and adolescents with chronic pain. The present ICC values were also greater than those reported in children and adolescents with cancer (0.22-0.68) [24] and sickle cell disease (0.30-0.45) [65]. The present greater ICC values may have resulted from the large percentage of female patients and their mothers being surveyed; however, this dyad gender effect has not been observed previously [16,54,66]. While still significant, the lower observed ICC of 0.48 for the PedsQL^TM^ parent-child Physical Health Summary Scores for 8-12 year olds may represent greater self-perception and self-awareness of physical limitations in the younger child.

In our clinical study sample and setting, patient age, patient gender, patient race, the intensity and duration of the patient’s pain, household income, parental marital status, and the parent’s own HRQoL were not significantly associated with the child-parent agreement about the patient’s HRQoL. Similarly, in an clinically established cohort of children with sickle cell disease, a logistic regression model did not identify patient age, family income, or the parent’s own HRQoL to be associated with a greater odds ratio for patient-parent discordance on the PedsQLTM Physical Health and Psychosocial Health Summary Scores [65]. In contrast, using the Juvenile Arthritis Quality of Life Questionnaire (JAQQ) and a linear regression model, greater child-parent agreement about quality of life in juvenile idiopathic arthritis (JIA) patients has been observed with longer disease duration [56]. Furthermore, in chronically ill adolescents, patient age, educational level and type of education, parent’s educational level and several other disease-related factors influenced child-parent disagreement on the KIDSCREEN-10 and DISABKIDS [67].

What is the clinician managing pediatric chronic pain to do when faced with such cases of poor agreement between patient self-reported versus parent proxy-reported HRQoL? While ostensibly preferable, adhering to recommendations to focus primarily on patients’ self-reports of such health information [3,68] is not prudent if it leads to a loss in parental confidence in the clinician and disaffection [69-71]. A more practical approach to managing pediatric chronic pain includes the prompt, transparent yet tactful acknowledgement of such patient-parent discordance in perceived health status [72,73]. Older children and adolescents can then manage to solicit parental assistance without losing the opportunity to present their own symptom accounts [74]. Even if delivered diplomatically, it has been our experience that such a candid dialogue with the patient and parent is not always initially or ultimately well-received—resulting in the patient being lost to follow-up. Such candor can nevertheless foster greater insight and acceptance of the contribution of the psychosocial dynamics (e.g., promoting of a sickness model and persistent child dependency) within the child-parent dyad to the pediatric pain experience [75-77].

### Limitations

Limitations of our study include the relatively small sample size; however, it was comparable to that of other published pediatric chronic pain cohorts. A similar demographic and clinical profile has been observed during the last decade among patients referred to four other tertiary-care, multidisciplinary pediatric pain medicine clinics in the United States [78-81]. Given its setting in a tertiary care hospital and affiliated subspecialty clinic, the present findings may not be applicable to a primary care pediatric practice and patient population. Furthermore, given that in the present study, the HRQoL instruments were all completed in an outpatient clinic, no insight can be offered as to the effect of varying location of data collection (e.g., hospital ward, outpatient clinic, home, and classroom).

## Conclusions

In summary, there frequently is clinically significant variation between pediatric chronic pain patients’ self-report and their parents’ proxy-report of their child’s HRQoL. No conventional demographic or clinical variables were associated with child-parent agreement about such patients’ HRQoL. There does not appear to be a significant correlation between such pediatric patients’ and their parents’ own respective perceptions of their HRQoL. While whenever possible the pediatric chronic pain patient’s perspective should be directly solicited, equal attention and merit should be given to the parent’s proxy-report of HRQoL. To do otherwise will obviate the opportunity to use any discordance as the basis for a therapeutic discussion about the contributing dynamic with in parent-child dyad. Further study is needed of the complex role parents, siblings and peers, in addition to the classroom setting and the community, likely play in the self-perceived health-related quality of life of a child or adolescent with a chronic pain condition.

## Abbreviation

HRQoL: Health-related quality of life.

## Competing interests

All three authors declare that they have no financial, consultant, institutional or other relationships that resulted in bias or a conflict of interest in the conducting or reporting this study. The authors have no competing interests.

## Authors’ contribution

All three authors were involved in drafting the article or critically revising it for important intellectual content, and all authors approved the final version to be submitted for publication. As the study principal investigator, TRV had full access to all of the data in the study, and he takes responsibility for the integrity of the data and the accuracy of the data analysis. TRV and CLB participated in study conception and design; CLB was responsible for acquisition of data; and TRV and GM performed the analysis and interpretation of data. All authors read and approved the final manuscript.

## Authors’ information

TRV is the Vice Chair of Pain Medicine in the Department of Anesthesiology at the University of Alabama at Birmingham. TRV has practiced pediatric pain medicine since 1989. He holds an MPH in Clinical Outcomes.

CLB is a nurse practitioner at the Children’s Hospital of Alabama. CLB has extensive experience in pediatric health care, including in pediatric stem cell transplant, pain medicine, and rheumatology.

GM is the Vice Chair of Epidemiology in the School of Public Health at the University of Alabama at Birmingham. GM has published extensively in health and quality of life outcomes.
